# Effects of DPP4 Inhibition on Inflammation and Oxidative Stress in Experimental Gestational Diabetes Mellitus

**DOI:** 10.3390/ijms27188303

**Published:** 2026-09-18

**Authors:** Öznur Tufan Akarslan, Ayşe Er

**Affiliations:** Department of Pharmacology and Toxicology, Faculty of Veterinary Medicine, Selcuk University, 42130 Konya, Turkey; oznrtfn@gmail.com

**Keywords:** gestational diabetes, placenta, cytokine, vildagliptin

## Abstract

Gestational diabetes mellitus (GDM) is a common pregnancy complication, with inflammation and oxidative stress playing a significant role in its pathogenesis. Therefore, this study evaluated the effects of vildagliptin on metabolic, hormonal, inflammatory, and oxidative stress parameters in an STZ-induced GDM model. Rats were divided into five groups: control, GDM, GDM + V10, GDM + V20 and GDM + V30. High blood glucose levels were observed in the GDM group, while a slight decrease was observed in the vildagliptin groups. While GLP-1 levels decreased in the GDM group, they increased in the GDM + V20 and GDM + V30 groups. Overall, an increase in pro-inflammatory cytokines and a decrease in anti-inflammatory cytokines were observed in the GDM group, while vildagliptin administration reversed these effects. Among the vildagliptin groups, IL-10 was found to be lowest and IL-6 highest in the GDM + V20 group. Although fluctuations were generally observed in oxidative stress markers, the highest MDA levels and the lowest GSH levels were found in the GDM + V20 group. In the GDM group, FW and PE were lower, and PW was higher. In contrast, FW and PE were higher, and PW was lower in the GDM + V30 group than in the GDM group. The best results were obtained at a dose of 30 mg/kg; however, further research is needed to confirm this effect.

## 1. Introduction

Gestational diabetes mellitus (GDM) is defined as glucose intolerance or hyperglycemia first detected or developing during pregnancy in women without a prior diagnosis of diabetes mellitus [1,2]. It is a leading cause of maternal and neonatal morbidity and mortality worldwide and is recognized as a major public health concern [3,4].

Pregnancy is accompanied by substantial metabolic adaptations that change across gestation to ensure an adequate nutrient supply to the developing fetus [5]. During the second and third trimesters, increasing concentrations of human placental lactogen, placental growth hormone, cortisol, and prolactin progressively reduce maternal insulin sensitivity, thereby limiting glucose uptake by maternal skeletal muscle and adipose tissue and increasing glucose availability to the fetus [6,7]. In late pregnancy, enhanced lipolysis and elevated circulating free fatty acids further impair insulin signaling and may promote mitochondrial reactive oxygen species (ROS) production, thereby linking insulin resistance with oxidative stress [8,9]. Under physiological conditions, pancreatic β-cells compensate for this progressive insulin resistance by increasing insulin secretion and functional capacity. However, when β-cell compensation is insufficient to meet the increased insulin demand, maternal hyperglycemia and GDM develop [10].

Increased cytokine production associated with the expansion of placental and adipose tissues during pregnancy is further exacerbated in GDM, highlighting the critical role of inflammatory mechanisms in its pathogenesis [11]. The inflammatory response also profoundly impacts fetal development [12]. Notably, levels of the pro-inflammatory cytokines tumor necrosis factor alpha (TNFα) and interleukin 6 (IL-6) are significantly elevated in women with GDM [13,14], whereas those of anti-inflammatory cytokines IL-10 and IL-4, which normally counterbalance the pro-inflammatory effects of TNFα and IL-6, are significantly lower [15]. Thus, inflammatory homeostasis is disrupted in GDM, potentially impairing placental function and contributing to the pathogenesis of adverse pregnancy outcomes.

Oxidative stress arises from an imbalance between ROS and antioxidant defense systems that favors pro-oxidants [16]. Physiological ROS production increases during pregnancy due to elevated metabolic activity and oxygen consumption, and this increase becomes more pronounced in GDM when combined with hyperglycemia [17]. Antioxidant defense systems, including superoxide dismutase (SOD), catalase (CAT), and glutathione (GSH), play critical roles in limiting oxidative damage [18]. Therefore, the reduced antioxidant capacity and increased levels of malondialdehyde (MDA), a marker of lipid peroxidation, observed in GDM suggest that oxidative stress is a key mechanism underlying its pathogenesis [19].

Inflammation and oxidative stress are recognized as two interrelated mechanisms in the pathophysiology of GDM. During the inflammatory response, increased production of pro-inflammatory cytokines [20] may enhance ROS production [21], leading to oxidative stress, while increased oxidative stress can activate inflammatory signaling pathways, thereby promoting cytokine production. Thus, a self-perpetuating inflammation–oxidative stress cycle is established [11,21].

Dietary modification and lifestyle interventions are the first-line approaches to managing GDM [22]. When adequate glycemic control cannot be achieved, insulin is recommended as the first-line pharmacological treatment [2]. Although oral hypoglycemic agents such as metformin [23] and glibenclamide [24] are used in certain circumstances, concerns about their safety during pregnancy and long-term effects remain. Therefore, investigating novel therapeutic strategies targeting the underlying pathophysiological mechanisms of GDM is of considerable importance.

The incretin system plays an important role in glucose homeostasis by regulating glucose-dependent insulin secretion. Following nutrient intake, glucagon-like peptide-1 (GLP-1) and glucose-dependent insulinotropic polypeptide (GIP) are secreted from enteroendocrine L cells and K cells of the intestine, respectively. GLP-1 stimulates glucose-dependent insulin secretion, suppresses glucagon release, and delays gastric emptying, whereas GIP predominantly enhances glucose-dependent insulin secretion from pancreatic β-cells [25,26]. Both incretin hormones are rapidly inactivated by dipeptidyl peptidase-4 (DPP4), thereby limiting the duration of their biological effects [27]. Emerging evidence suggests that dysregulation of this incretin–DPP4 axis may contribute to insufficient β-cell compensation in GDM, as reduced postprandial GLP-1 responses and increased DPP4 activity have been reported in affected women [28].

By preventing incretin degradation, DPP4 inhibitors increase the circulating levels of GLP-1 and GIP and prolong their biological effects [26]. Moreover, markedly elevated placental DPP4 activity in the maternal circulation has been reported in women with GDM [29]. Consequently, interest in the potential therapeutic application of DPP4 inhibitors in GDM has increased in recent years [30]. Therefore, this study aimed to evaluate the effects of vildagliptin, a DPP4 inhibitor, on placental inflammation and oxidative stress in an experimental rat model of GDM.

## 2. Results

### 2.1. Effects of Vildagliptin on Metabolic and Biochemical Parameters

**Figure 1:**
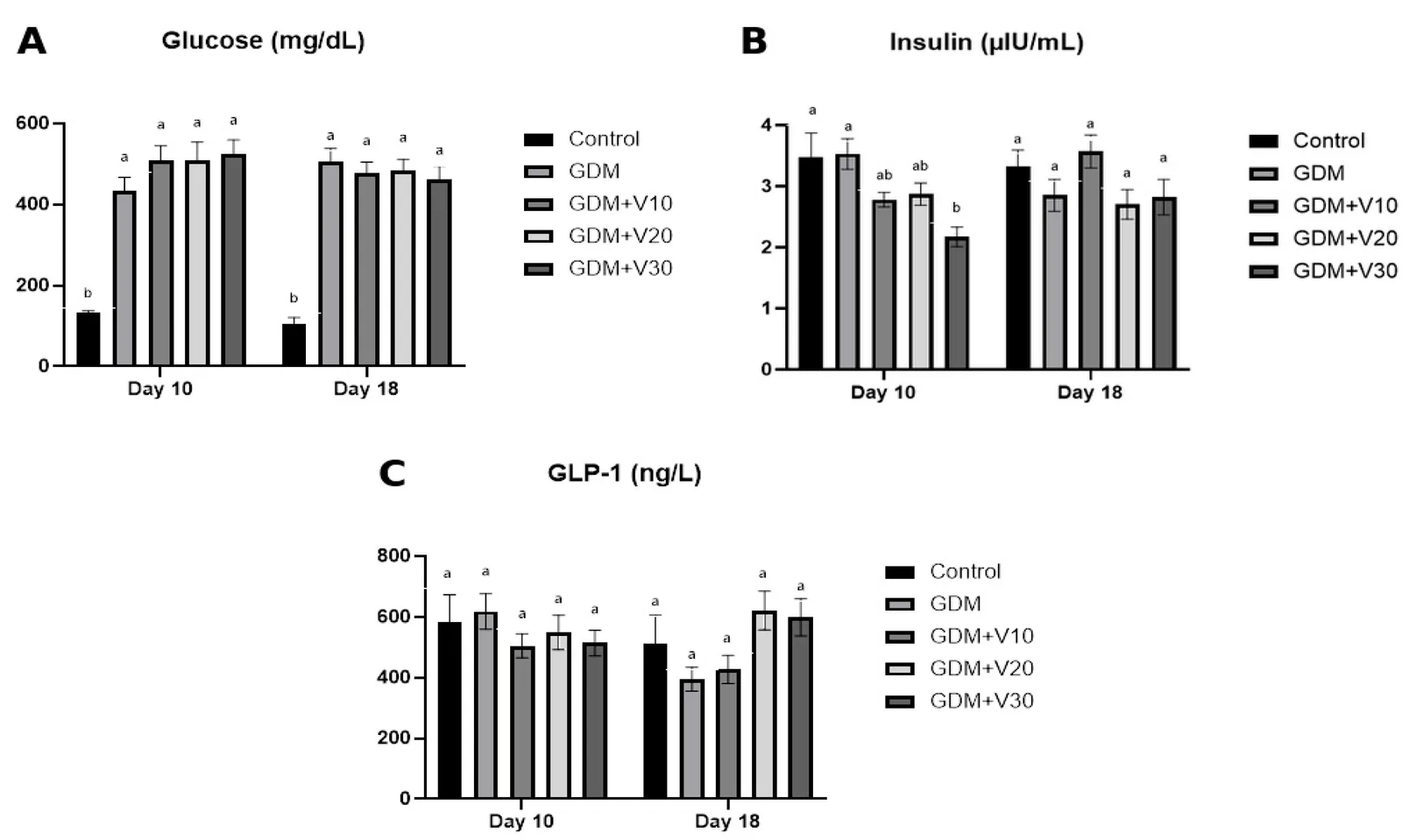
Effects of vildagliptin on blood glucose, insulin, and GLP-1 levels on gestational days 10 and 18 in gestational diabetes mellitus. Plasma glucose (**A**), insulin (**B**), and glucagon-like peptide-1 (GLP-1) (**C**) levels measured on gestational days 10 and 18. Data are presented as mean ± SE. Different lowercase letters (a,b) indicate statistical groupings determined by one-way ANOVA followed by Tukey’s post hoc multiple-comparison test. Groups sharing at least one letter are not significantly different, whereas groups with no letter in common differ significantly (*p* < 0.05). Control, *n* = 5; GDM, *n* = 8; GDM + V10, *n* = 10; GDM + V20, *n* = 9; and GDM + V30, *n* = 9. GDM, gestational diabetes mellitus; V10, vildagliptin 10 mg/kg; V20, vildagliptin 20 mg/kg; V30, vildagliptin 30 mg/kg.

On gestational day 10, blood glucose levels were significantly higher in the GDM group than in the control group (*p* < 0.05) (Figure 1A). This elevation persisted on gestational day 18, whereas blood glucose levels were not significantly lower in the GDM + V10, GDM + V20, and GDM + V30 groups than in the GDM group (*p* > 0.05) (Figure 1A). On gestational day 10, insulin levels were significantly lower in the GDM + V30 group than in both the control and GDM groups (*p* < 0.05) (Figure 1B). Although there was no statistically significant difference, GLP-1 levels decreased in the GDM group on day 18 of pregnancy, while this decrease was prevented, particularly in the GDM + V20 and GDM + V30 groups (Figure 1C). No statistically significant difference was detected between the GDM group and the control group, or between the vildagliptin-administered groups and the GDM group, in terms of AST, BUN and creatinine levels. No statistically significant differences were observed between the groups regarding ALT, cholesterol, triglyceride, HDL, and LDL levels (*p* > 0.05). Nevertheless, AST levels were highest in the GDM + V20 group (Table 1).

### 2.2. Effects of Vildagliptin on Placental Inflammatory Parameters

**Figure 2:**
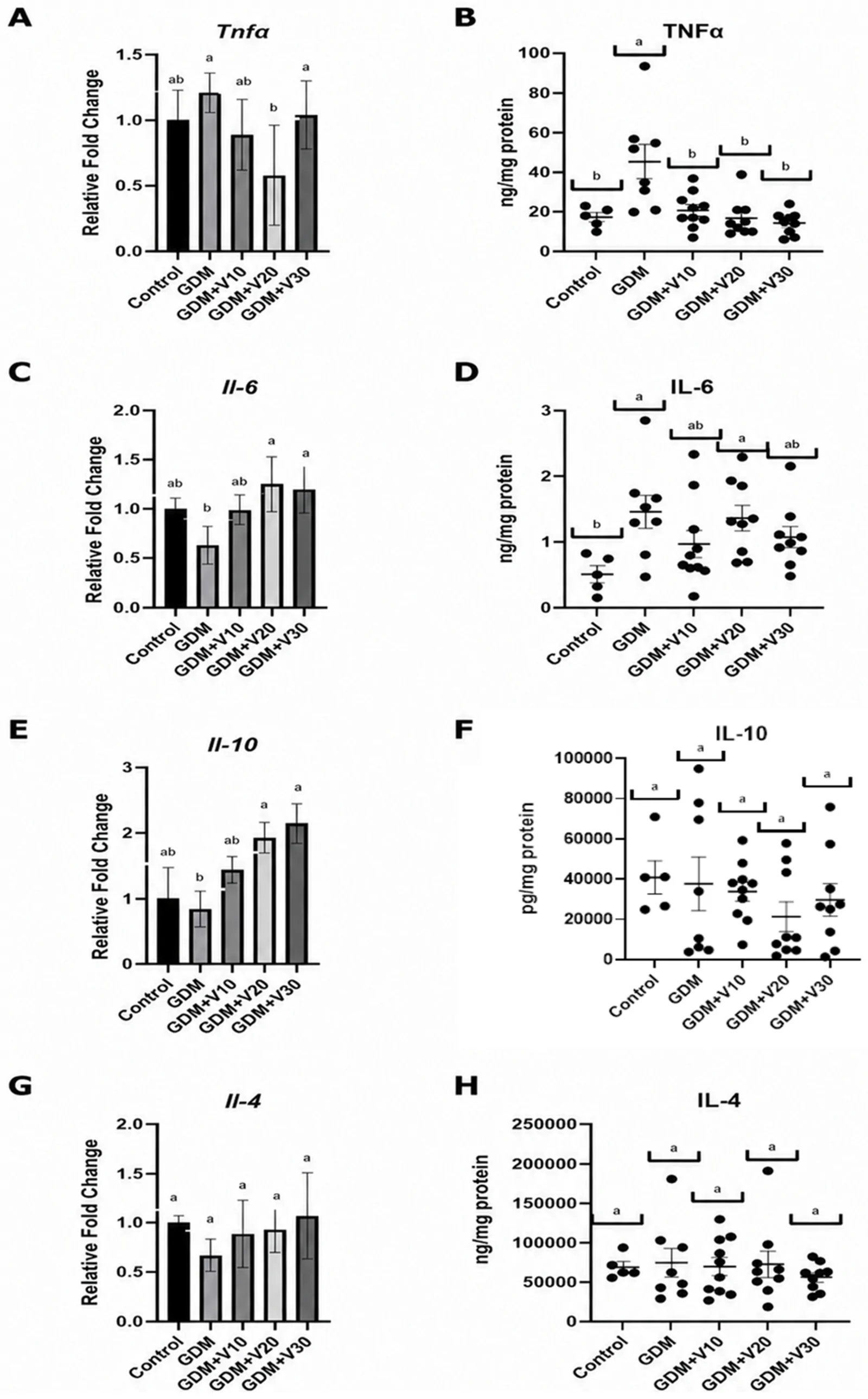
Effects of vildagliptin on placental mRNA expression and protein levels of pro-inflammatory and anti-inflammatory cytokines in gestational diabetes mellitus. Relative mRNA expression levels of *Tnfα* (**A**), *Il-6* (**C**), *Il-10* (**E**), and *Il-4* (**G**), and protein levels of TNFα (**B**), IL-6 (**D**), IL-10 (**F**), and IL-4 (**H**) in placental tissues. Data are presented as mean ± SE. Different lowercase letters (a,b) indicate statistical groupings determined by one-way ANOVA followed by Tukey’s post hoc multiple-comparison test. Groups sharing at least one letter are not significantly different, whereas groups with no letter in common differ significantly (*p* < 0.05). Group sample sizes were as follows: control (*n* = 5), GDM (*n* = 8), GDM + V10 (*n* = 10), GDM + V20 (*n* = 9), and GDM + V30 (*n* = 9). GDM, gestational diabetes mellitus; V10, vildagliptin 10 mg/kg; V20, vildagliptin 20 mg/kg; V30, vildagliptin 30 mg/kg; TNF α, tumor necrosis factor α; IL, interleukin.

Regarding the pro-inflammatory cytokines, *Tnfα* mRNA levels (Figure 2A) were not significantly higher in the GDM group than in the control group, whereas *Il-6* mRNA levels (Figure 2C) were not significantly lower (*p* > 0.05). In contrast, protein levels of both TNFα (Figure 2B) and IL-6 (Figure 2D) were significantly higher in the GDM group than in the control group (*p* < 0.05). Additionally, *Tnfα* mRNA and protein levels were significantly lower in the GDM + V10, GDM + V20, and GDM + V30 groups than in the GDM group. In contrast, *Il-6* mRNA levels were higher in the GDM + V10 (*p* > 0.05), GDM + V20 (*p* < 0.05), and GDM + V30 (*p* < 0.05) groups than in the GDM group, whereas IL-6 protein levels were significantly lower (*p* > 0.05).

Regarding the anti-inflammatory cytokines, *Il-10* (Figure 2E) and *Il-4* (Figure 2G) mRNA levels were not significantly lower in the GDM group than in the control group. IL-10 protein levels (Figure 2F) were not significantly lower in the GDM group than in the control group, whereas IL-4 protein levels (Figure 2H) were not significantly higher. Although *Il*-4 and *Il-10* mRNA levels were higher in the GDM + V10, GDM + V20, and GDM + V30 groups than in the GDM group, the difference was only significant for IL-10 levels in the GDM + V20 and GDM + V30 groups (*p* < 0.05). However, IL-4 and IL-10 protein levels did not differ significantly between the GDM + V10, GDM + V20, and GDM + V30 groups and the GDM group (*p* > 0.05). Nevertheless, IL-10 protein levels were lowest in the GDM + V20 group, and IL-4 protein levels were lowest in the GDM + V30 group.

### 2.3. Effects of Vildagliptin on Placental Oxidative Stress and Antioxidant Parameters

**Figure 3:**
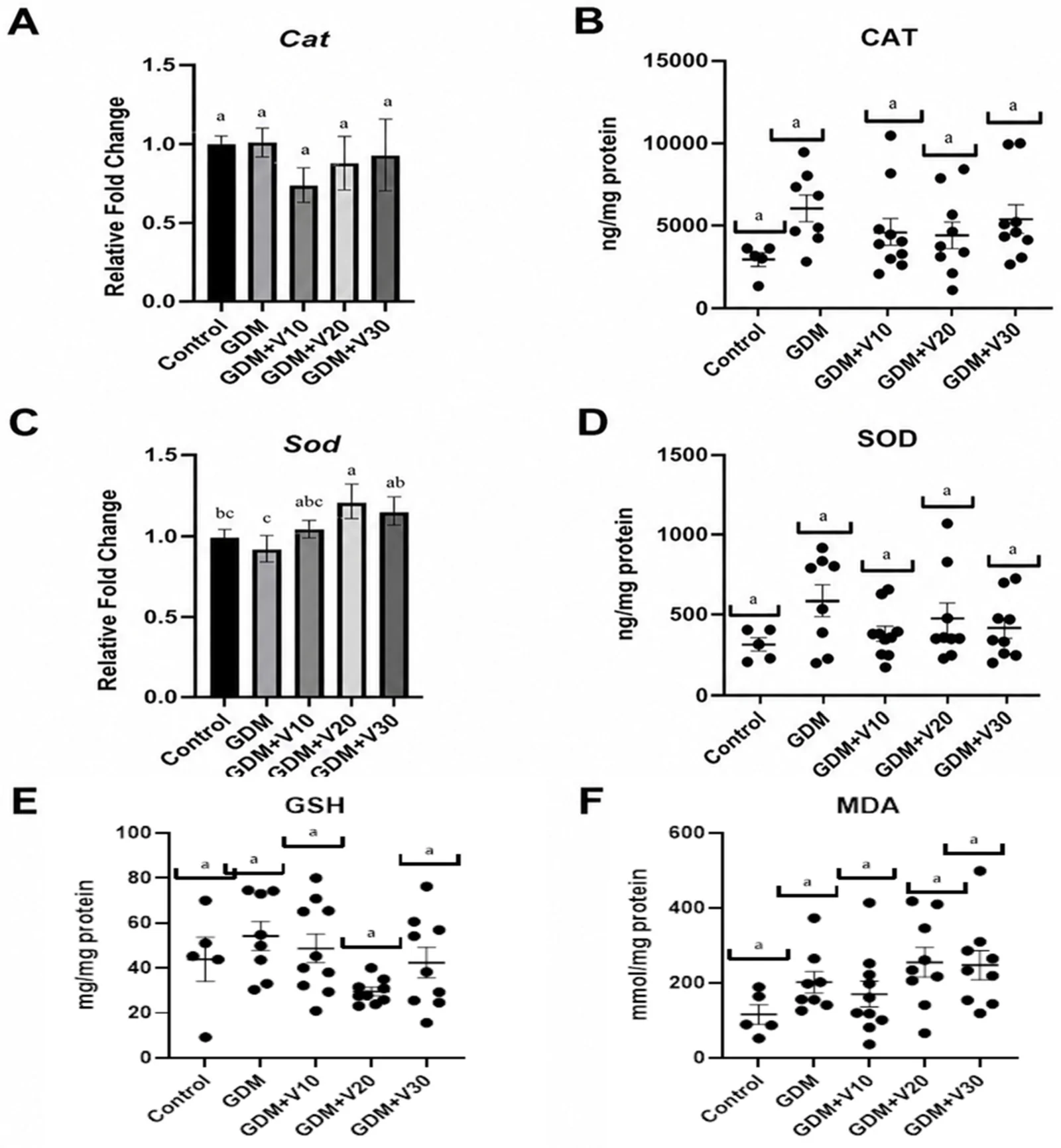
Effects of vildagliptin on placental antioxidant enzyme mRNA expression and oxidative stress marker levels in gestational diabetes mellitus. Relative mRNA expression (fold change) of *Cat* (**A**) and *Sod* (**C**), protein levels of CAT (**B**) and SOD (**D**), and levels of GSH (**E**) and MDA (**F**) in placental tissues. Data are presented as mean ± SE. Different lowercase letters (a,b,c) indicate statistical groupings determined by one-way ANOVA followed by Tukey’s post hoc multiple-comparison test. Groups sharing at least one letter are not significantly different, whereas groups with no letter in common differ significantly (*p* < 0.05). Group sample sizes were as follows: control (*n* = 5), GDM (*n* = 8), GDM + V10 (*n* = 10), GDM + V20 (*n* = 9), and GDM + V30 (*n* = 9). GDM, gestational diabetes mellitus; V10, vildagliptin 10 mg/kg; V20, vildagliptin 20 mg/kg; V30, vildagliptin 30 mg/kg; CAT, catalase; SOD, superoxide dismutase; GSH, glutathione; MDA, malondialdehyde.

Regarding the oxidative stress markers, *Cat* mRNA levels (Figure 3A), CAT and SOD protein levels (Figure 3B,D), MDA (Figure 3F), and GSH levels (Figure 3E) did not differ significantly across groups (*p* > 0.05). Compared with the GDM group, *Sod* mRNA levels (Figure 3C) were significantly higher in the GDM + V20 and GDM + V30 groups (*p* < 0.05) and were not significantly higher in the GDM + V10 group. MDA levels were highest, and GSH levels were lowest in the GDM + V20 group.

### 2.4. Effects of Vildagliptin on Fetoplacental Outcomes

Regarding fetal and placental growth parameters, although mean fetal weight (FW) and mean placental weight (PW) did not differ significantly across groups, FW was lower (Hedges’ g = −0.50), and PW was higher (Hedges’ g = 0.92) in the GDM group than in the control group. In contrast, FW was higher (Hedges’ g = 0.06), and PW was lower (Hedges’ g = −0.49) in the GDM + V30 group than in the GDM group. Placental efficiency (PE) was not significantly lower in the GDM group (Hedges’ g = −1.10) than in the control group and not significantly higher in the GDM + V30 group (Hedges’ g = 0.53) than in the GDM group. In addition, PE was lowest in the GDM + V10 group (Table 2).

## 3. Discussion

GDM is a complex metabolic disorder with increasing prevalence, in which inflammation, oxidative stress, and alterations in the incretin system play important roles [31,32]. Moreover, DPP4, an enzyme involved in regulating insulin secretion and glucose homeostasis via its effects on the incretin system, has been proposed as an important biological target in the development of GDM and the maintenance of metabolic control [29,30]. Therefore, our study investigated the effects of the DPP4 inhibitor vildagliptin on biochemical, inflammatory, and oxidative stress parameters in an experimental rat model of GDM.

Our study showed that blood glucose levels were significantly higher in the GDM group than in the control group on both gestational days 10 and 18 (*p* < 0.05) (Figure 1A). These findings are consistent with previous studies [33,34] and indicate that the experimental rat model of GDM was successfully established. In this study, although a decrease in blood glucose levels was observed after vildagliptin administration, this change was determined to be not statistically significant. In several studies evaluating vildagliptin administration for 8–12 weeks at doses ranging from 1 to 20 mg/kg, statistically significant but limited decreases in blood glucose levels have been reported [35,36,37]. Therefore, although the decrease observed in the present study was not statistically significant, it is generally consistent with previously reported pharmacological effects of vildagliptin. Therefore, our findings do not suggest that vildagliptin lacks pharmacological activity; rather, the observed decrease may be due to insufficient statistical significance achieved under the experimental conditions of this study (experimental model, baseline glucose levels, physiological capacity of the incretin system, biological variability, etc.). Moreover, insulin levels were significantly lower in the GDM + V30 group than in both the control and GDM groups on gestational day 10 (Figure 1B). The fact that no significant difference was observed between the groups on gestational day 18, unlike at the initial sampling time, suggests that the effect of vildagliptin on insulin levels may vary depending on the changing metabolic conditions during the later stages of pregnancy. Previous studies using different experimental models of diabetes mellitus, including GDM, have reported inconsistent findings regarding insulin levels [34,35,38]. Although no statistically significant difference was found between the groups at either sampling time, it was observed that GLP-1 levels in the GDM + V20 and GDM + V30 groups reached higher values on gestational day 18, in contrast to the decrease observed in the GDM group. This finding can be considered a trend consistent with the known pharmacological effect of vildagliptin, which increases GLP-1 levels by reducing GLP-1 degradation through DPP4 inhibition. At the end of our study, the observed improvement in blood glucose levels in the vildagliptin groups suggests that the effects of vildagliptin on glycemic control may occur through multiple mechanisms.

In our study, fluctuations in AST, BUN, and creatinine levels were observed in the GDM group. Similar results were seen in the vildagliptin-administered groups, but AST levels were highest in the GDM + V20 group, which likely reflects placental oxidative stress and inflammation (Table 1). In contrast to the changes observed in AST, BUN, and creatinine, vildagliptin administration did not significantly affect ALT or the evaluated lipid parameters, including cholesterol, triglycerides, HDL, and LDL, indicating a limited effect on the general biochemical profile under the present experimental conditions. Overall, the lack of marked adverse effects on hepatic and renal function, as well as on lipid metabolism, in the vildagliptin-treated groups is consistent with previous studies [39,40,41,42].

Our study observed increases in the pro-inflammatory cytokines TNFα (mRNA and protein levels) and IL-6 (protein levels only), along with decreases in the anti-inflammatory cytokines IL-10 (mRNA and protein levels) and IL-4 (mRNA levels only) in the GDM group (Figure 2A,C,E,G), indicating the presence of placental inflammation and consistent with previous studies [15,43,44,45]. In the groups administered vildagliptin, the increase in *Tnfα* mRNA levels caused by GDM was reduced; this reduction was found to be statistically significant only in the GDM + V20 group. In contrast, a statistically significant decrease in TNFα protein levels was observed in all vildagliptin groups. In contrast, the lower *Il-6* mRNA levels despite elevated IL-6 protein levels in the GDM group may be explained by signal transducer and activator of transcription 3/suppressor of cytokine signaling-3-mediated negative feedback mechanisms involved in IL-6 signaling [46,47]. In the groups administered vildagliptin, the increase in IL-6 protein levels caused by GDM was reduced—most notably in the GDM + V10 and GDM + V30 groups—although the difference was not statistically significant. TNFα and IL-6 are key pro-inflammatory cytokines involved in the pathogenesis of GDM, and their suppression is considered crucial for reducing inflammation during treatment, as demonstrated in our study. While *Il-10* mRNA and protein levels decreased in the GDM group, a significant increase in mRNA expression was observed in the groups administered vildagliptin, particularly the GDM + V20 and GDM + V30 groups; however, this increase was not reflected in protein levels, where slight decreases were observed, with the exception of the GDM + V20 group. *Il-4* mRNA levels decreased in the GDM group, while they increased in the groups administered vildagliptin. In contrast, IL-4 protein levels were similar across all groups, with no significant difference observed. While TNFα and IL-6 levels were generally similar in the GDM + V10 and GDM groups, comparisons of the GDM + V20 and GDM + V30 groups suggest a stronger anti-inflammatory effect in the GDM + V30 group, as evidenced by greater reductions in TNFα and IL-6 protein levels and higher IL-10 protein levels (Figure 2B,D). Furthermore, the increases in *Il*-6 mRNA and protein levels in the GDM + V20 group may reflect drug-induced stress or tissue injury, consistent with the increased blood glucose, AST, SOD, and MDA levels, along with the decreased IL-10, CAT, and GSH levels observed in that group.

In our study, except for *Sod* mRNA levels, statistically non-significant increases in oxidative stress markers were detected in the GDM group, and non-significant fluctuations were observed between the vildagliptin groups. Therefore, it is considered that the potential effect of vildagliptin on oxidative stress may have remained limited under these experimental conditions or may not have been clearly reflected in the parameters evaluated. *Sod* mRNA levels were higher in the GDM + V10, GDM + V20, and GDM + V30 groups compared to the GDM group and reached statistical significance in the GDM + V20 and GDM + V30 groups (Figure 3C). In addition, MDA levels were highest, and GSH levels were lowest in the GDM + V20 group (Figure 3E,F). Conflicting findings have been reported in the literature regarding placental SOD and CAT levels, with studies demonstrating both increases and decreases in these antioxidant enzymes. In contrast, most therapeutic interventions have been associated with increased antioxidant enzyme levels [48,49]. Similarly, while placental MDA levels have consistently been reported to increase, findings regarding GSH levels have been mixed [50,51]. The administration of different therapeutic agents has been shown to suppress oxidative stress by decreasing MDA levels and increasing GSH levels, thereby enhancing antioxidant capacity [33,48,49]. Oxidative stress is considered one of the principal mechanisms underlying the pathophysiology of GDM and results from an imbalance between increased ROS production and antioxidant defense systems [17]. In our study, the lower *Sod* mRNA levels observed in the GDM group may be attributed to depletion or transcriptional suppression of the antioxidant defense system in response to increased oxidative stress. Moreover, higher SOD and CAT protein levels may indicate a compensatory activation of antioxidant defense mechanisms in response to increased oxidative stress. The decreases in SOD and CAT protein levels observed following vildagliptin treatment may be attributable to its regulatory effects on oxidative stress, thereby reducing the oxidative burden. Although the low GSH level observed in the GDM + V20 group may explain the increased MDA level in that group, the lack of a corresponding increase in CAT protein levels despite elevated SOD protein levels suggests that ROS were not sufficiently detoxified, leading to more pronounced oxidative damage in the placenta. In the GDM + V30 group, despite MDA levels remaining comparable to those in the GDM + V20 group, the increases in GSH and CAT protein levels indicate a more effective compensatory antioxidant response against oxidative damage in the placenta. This adaptive response may also explain why PE was highest in the GDM + V30 group.

In our study, FW, PW, and PE were calculated for each dam to evaluate fetal and placental growth parameters. Compared with the control group, the GDM group had non-significantly lower FW and PE and higher PW. Moreover, compared with the GDM group, the GDM + V30 group had higher FW, lower PW, and thus higher PE (Table 2). Previous studies using experimental models of GDM have reported both decreases and increases in fetal and placental weights, which were reversed following treatment with different therapeutic agents [33,34,43,45,51]. Studies evaluating PE have also demonstrated that this parameter is lower in the GDM group than in the control group [52,53]. In our study, despite compensatory placental enlargement, reduced placental functional efficiency appeared to suppress intrauterine growth, ultimately resulting in decreased PE. Moreover, despite the elevated MDA levels observed in the GDM + V30 group, the increase in PE suggests that oxidative stress may have triggered compensatory adaptive responses in the placenta. In this context, the antioxidant and anti-inflammatory effects of vildagliptin at this dose may have improved placental function, thereby supporting fetal growth.

In our study, it was observed that the effects of vildagliptin on most of the evaluated parameters did not change linearly with increasing doses. Although more pronounced responses were observed for certain parameters at higher doses, the highest dose did not appear to offer any additional advantage across all parameters compared to lower doses. Similarly, the finding reported by Zhang et al. [36] that the reduction in blood glucose levels was more pronounced at a dose of 10 mg/kg compared to 20 mg/kg supports the notion that the biological effects of vildagliptin are not always proportional to dose increases. This non-linear response may be attributed to the fact that, once a sufficient level of DPP4 inhibition is achieved, higher doses of vildagliptin exert only a limited additional effect on the incretin-mediated response. Nevertheless, while varying dose–response patterns were observed across individual biochemical and molecular parameters, the collective assessment of these parameters allowed for a more holistic evaluation of vildagliptin’s overall placental impact. Consequently, it is suggested that the efficacy of vildagliptin should be assessed not merely through changes in individual parameters resulting from dose increases, but rather through a comprehensive evaluation of various endpoints.

## 4. Materials and Methods

### 4.1. Animals

All study procedures were approved by the Local Ethics Committee for Animal Experiments of the Experimental Medicine Application and Research Center at Selcuk University (approval no. 2023-53). This study involved 46 female Wistar Albino rats (12 weeks old) with comparable ages and body weights. Prior to inclusion in the study, all animals underwent health examinations to ensure their suitability for the experiment. Throughout the experimental period, the animals had ad libitum access to standard feed and water. Environmental conditions were maintained under controlled conditions at a temperature of 22 ± 2 °C, a 12 h light/12 h dark cycle, and a humidity of 55 ± 5%. The ARRIVE checklist was used to assess the reporting of the animal experiment presented in this manuscript.

### 4.2. Experimental Design and Treatments

**Figure 4:**
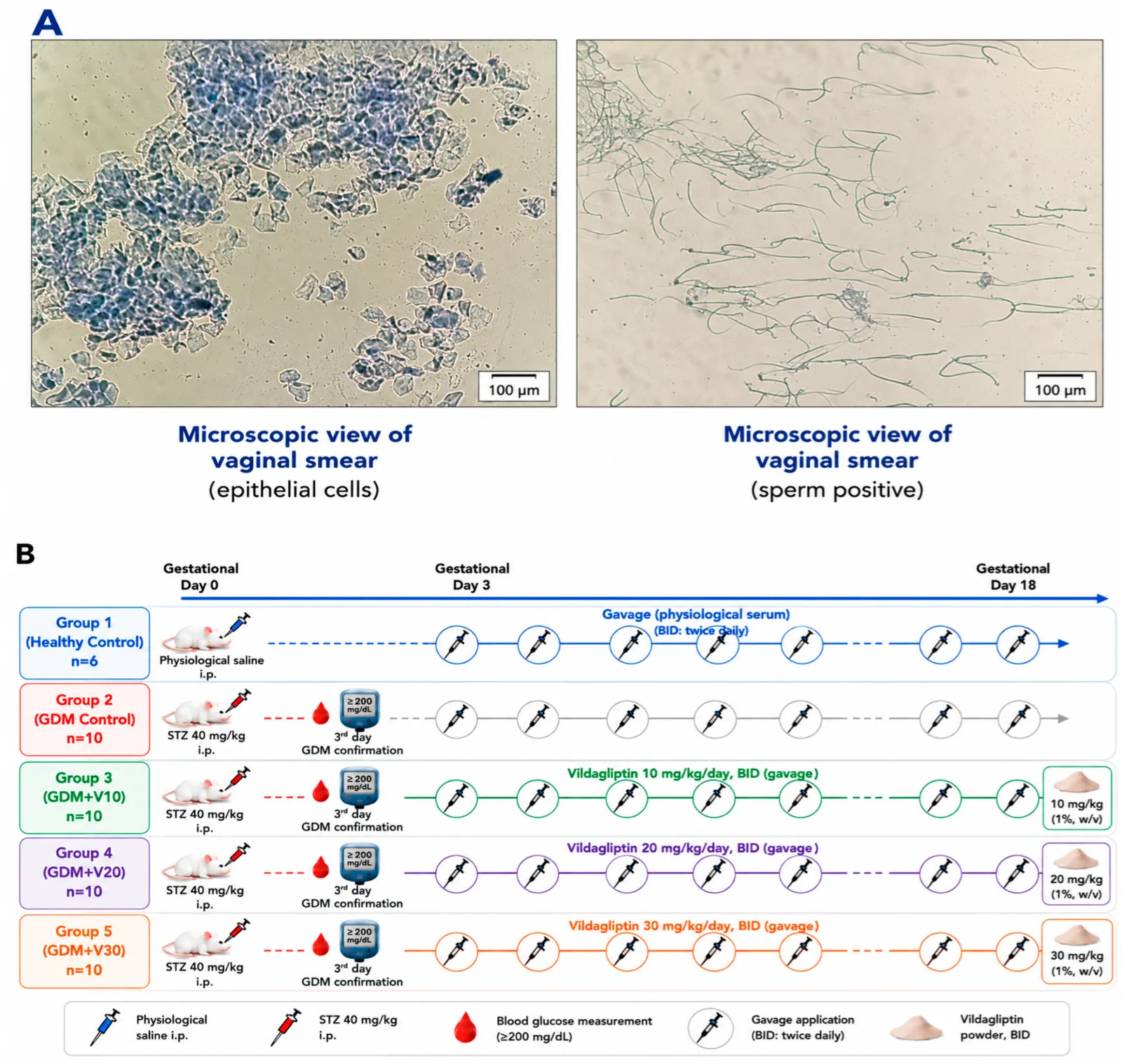
Representative microscopic images of vaginal smears obtained using a 20× objective: epithelial cells and sperm-positive vaginal smear (**A**); experimental design and applications (**B**).

Vaginal smears were collected from female rats daily to monitor the estrous cycle. Female rats in the estrus phase were mated with males at a female:male ratio of 2:1 per cage. The day on which sperm was first detected in the vaginal smear was designated as gestational day 0 (Figure 4A). To prepare the sodium citrate buffer, 0.1 M sodium citrate solution (Tekkim Kimya, Bursa, Türkiye) and 0.1 M citric acid solution (Tekkim Kimya, Bursa, Türkiye) were separately prepared in 50 mL of distilled water and then mixed until the pH reached 4.5.Streptozotocin (STZ, Sigma-Aldrich, Taufkirche, Germany) was freshly dissolved in the sodium citrate buffer at a final concentration of 20 mg/mL immediately before administration. Experimental GDM was induced in all rats except those in the control group (*n* = 6) by a single intraperitoneal injection of 2% STZ (40 mg/kg).

Rats with blood glucose levels ≥ 200 mg/dL on gestational day 3 were considered to have GDM [54,55]. These rats were randomly allocated to four groups (*n* = 10/group): GDM, GDM + V10 (vildagliptin at 10 mg/kg/day; [36]), GDM + V20 (vildagliptin at 20 mg/kg/day; [36]), and GDM + V30 (vildagliptin at 30 mg/kg/day; [56]). Vildagliptin was administered orally twice daily from gestational day 3 to gestational day 18. Rats in the control group received a single intraperitoneal injection of physiological saline on gestational day 0 and physiological saline by oral gavage from gestational day 3 to gestational day 18 (Figure 4B). During the experimental period, five animals died [control (*n* = 1), GDM (*n* = 2), GDM + V20 (*n* = 1), and GDM + V30 (*n* = 1)], resulting in final group sizes of control (*n* = 5), GDM (*n* = 8), GDM + V10 (*n* = 10), GDM + V20 (*n* = 9), and GDM + V30 (*n* = 9).

### 4.3. Sample Collection

Gestational day 10 was selected as an intermediate time point to evaluate the metabolic response during vildagliptin treatment, whereas gestational day 18 was selected to assess these parameters at the end of the treatment period. On gestational day 10, blood samples were collected from the retro-orbital sinus under ether anesthesia to measure blood glucose, GLP-1, and insulin levels. On gestational day 18, rats were anesthetized with thiopental sodium (70 mg/kg, intraperitoneally) [57], and intracardiac blood samples were collected for serum and plasma analyses. The uterus was carefully removed, and the fetuses and placentas were collected for evaluation. Placental tissues and serum/plasma samples were stored at −80 °C until analysis.

### 4.4. Biochemical Parameters

In the blood samples collected on gestational days 10 and 18, blood glucose was measured using an autoanalyzer (Dirui CS-4000, Changchun, China), and insulin (catalog no.: E0707Ra) and GLP-1 (catalog no.: E0719Ra) were measured using commercially available enzyme-linked immunosorbent assay (ELISA) kits (Bioassay Technology Laboratory, Shanghai, China), whereas alanine aminotransferase, aspartate aminotransferase (AST), blood urea nitrogen (BUN), creatinine, total cholesterol, triglycerides, high-density lipoprotein (HDL), and low-density lipoprotein (LDL) were measured only in samples collected on gestational day 18. Placental tissue homogenates were prepared, and TNFα (catalog no.: E0764Ra), IL-4 (catalog no.: E0133Ra), IL-6 (catalog no.: E0135Ra), IL-10 (catalog no.: E0108Ra), SOD (catalog no.: E0168Ra), CAT (catalog no.: E0869Ra), MDA (catalog no.: E0156Ra), and GSH (catalog no.: E1101Ra) were measured using commercially available ELISA kits (Bioassay Technology Laboratory, Shanghai, China). All ELISAs were read using an ELISA reader (Lambda Scan 200; BioTek Instruments Inc., Winooski, VT, USA). The measurements in the placenta were normalized to the total protein (catalog no.: SH0292-2) content determined by ELISA in the corresponding samples and expressed as mg of protein.

### 4.5. Molecular Analyses

#### 4.5.1. RNA Isolation and Reverse Transcription

Total RNA was isolated from the placental tissue samples using commercial TRI Reagent (Sigma-Aldrich, St. Louis, MO, USA) according to the manufacturer’s instructions. Briefly, approximately 50 mg of placental tissue was homogenized in approximately 1 mL of TRI Reagent. Next, the homogenate was mixed with 300 μL of chloroform and centrifuged. Then, the supernatant was extracted and mixed with 500 μL of isopropanol. The RNA pellet was then washed three times with cold ethanol (70%, 70%, and 99%, respectively), air-dried, and dissolved in 40 μL diethyl pyrocarbonate (DEPC)-treated water. RNA quality and concentration were assessed using a spectrophotometer (NanoDrop; Thermo Fisher Scientific, Waltham, MA, USA), with DEPC-treated water used as the blank. Next, RNA samples (1 μg/10 μL) were electrophoresed on a 1% agarose gel and visualized with ethidium bromide under a UV transilluminator to evaluate band quality. RNA samples with A260/A280 ratios between 1.8 and 2.0 and A260/A230 ratios between 2.0 and 2.2 were adjusted to 1 μg/2.5 μL using DEPC-treated water. Then, genomic DNA was removed by DNase I treatment (Thermo Fisher Scientific, Waltham, MA, USA). Finally, the RNA was reverse transcribed using the iScript™ cDNA Synthesis Kit (Bio-Rad, Hercules, CA, USA) in a total reaction volume of 10 μL, and the synthesized complementary DNA (cDNA) was stored at −20 °C until needed.

#### 4.5.2. Real-Time Quantitative PCR (RT-qPCR)

Primer sequences for actin beta (*Actb*/*β-actin*), *Tnfα*, *Il-10*, *Il-6*, *Il-4*, *Cat*, and *Sod* were obtained from the literature and then verified using the Primer-BLAST tool (NCBI, available online: https://www.ncbi.nlm.nih.gov/tools/primer-blast/, accessed on 20 January 2024) to ensure primer specificity and to confirm that the primers neither formed dimers nor amplified unintended regions of the rat genome. The primers were then commercially synthesized (Bioligo, Ankara, Turkey) (Table 3). Lyophilized primers were dissolved in nuclease-free water to a concentration of 100 pmol/μL. Small amounts of cDNA from all samples were pooled in an Eppendorf tube, and primer efficiencies were estimated using serial dilutions of the pooled cDNA.

The levels of the target genes in the synthesized cDNA were measured by RT-qPCR using gene-specific primers. Each reaction consisted of 5 μL of SYBR Green Master Mix (Bio-Rad, Hercules, CA, USA), 100 pmol of the forward and reverse primers, 2 μL of cDNA, and nuclease-free water to a final volume of 10 μL. The thermal cycling protocol consisted of an initial denaturation/enzyme activation step at 95 °C for 5 min, followed by 40 cycles of denaturation at 95 °C for 10 s, annealing at 60 °C for 30 s, and extension at 72 °C for 30 s. Melt curve analysis was performed after amplification from 65 °C to 95 °C in increments of 0.2–0.5 °C, and amplification specificity was confirmed by the presence of a single peak. All RT-qPCRs were performed in triplicate. The obtained data were recorded as quantification cycle (Cq) values. The Cq values for the target genes were normalized to those of *β-actin* (a housekeeping gene), and fold changes were estimated using the 2^−ΔΔCt^ method [58].

**Table 3:** Primer sequences, amplicon sizes, and accession numbers used in RT-qPCR.

Gene Symbol	Primer Sequence (5′-3′)	Product Size (bp)	Accession	References
*Actb*-F	5′GGAGATTACTGCCCTGGCTCCTA3′	150	NM_031144.3	[59]
*Actb*-R	5′GACTCATCGTACTCCTGCTTGCTG3′			
*Tnfα*-F	5′ATGGGCTCCCTCTCATCAGT3′	106	NM_012675.3	[60]
*Tnfα*-R	5′GCTTGGTGGTTTGCTACGACG3′			
*Il10*-F	5′CAGACCCACATGCTCCGAGA3′	141	XM_006249712.4	[61]
*Il10*-R	5′CAAGGCTTGGCAACCCAAGTA3′			
*Il6*-F	5′TCTGTTGTGGGTGGTATCCTCTG3′	104	NM_012589.2	[62]
*Il6*-R	5′TCCAGCCAGTTGCCTTCTTG3′			
*Il4*-F	5′TGCACCGAGATGTTTGTACC3′	124	NM_201270.1	[63]
*Il4*-R	5′GGATGCTTTTTAGGCTTTCC3′			
*Sod*-F	5′AAGCGGTGAACCAGTTGTG3′	182	NM_017050.1	[64]
*Sod*-R	5′CCAGGTCTCCAACATGCC3′			
*Cat*-F	5′ATTGCCGTCCGATTCTCC3′	155	NM_012520.2	[65]
*Cat*-R	5′CCAGTTACCATCTTCAGTGTAG3′			

### 4.6. Assessment of Fetal and Placental Growth Parameters

The number of fetuses was recorded for each rat, and individual fetal and placental weights were measured. FW for each rat was calculated by dividing the total fetal weight by the number of fetuses. PW was calculated by dividing the total placental weight by the number of placentas. PE was calculated as the ratio of mean fetal weight to mean placental weight for each rat.

### 4.7. Statistical Analysis

All statistical analyses were performed using SPSS (version 22.0; IBM Corp., Armonk, NY, USA), and *p*-values < 0.05 were considered statistically significant. In the RT-qPCR analysis, fold changes are presented as the mean ± standard error (SE). Gene expression and biochemical parameters were compared between groups using one-way analysis of variance (ANOVA) followed by post hoc Tukey’s tests if significant.

## 5. Conclusions

Experimental GDM was characterized by marked hyperglycemia accompanied by alterations in placental inflammatory and oxidative stress-related parameters, indicating disruption of the inflammatory/oxidative balance. The present study demonstrated that vildagliptin exerted differential effects on placental inflammatory and oxidative stress-related alterations in experimental GDM, despite its limited effect on glycemic control. Vildagliptin treatment modulated inflammatory and antioxidant parameters, with the 30 mg/kg dose generally showing the most favorable effects, particularly in maintaining the inflammatory/oxidative balance and placental function. The higher PE observed at this dose further suggests a potential improvement in fetoplacental outcomes. However, the persistence of marked hyperglycemia despite treatment indicates that the beneficial effects of vildagliptin in this experimental model may not be primarily attributable to glycemic control. Moreover, the relatively small sample size and the use of an STZ-induced experimental model should be considered when interpreting these findings. Further studies using different experimental models, larger sample sizes, and additional mechanistic approaches are needed to clarify the pathways underlying these effects and to determine their potential translational relevance to GDM.

## Figures and Tables

**Table 1:** Effects of vildagliptin on serum biochemical parameters in gestational diabetes mellitus.

Parameter	Control	GDM	GDM + V10	GDM + V20	GDM + V30
ALT U/L	68.80 ± 8.58	108.75 ± 9.57	113.50 ± 11.56	127.11 ± 20.04	190.88 ± 79.35
AST U/L	138.6 ± 10.26 ^b^	188.25 ± 13.58 ^ab^	191.0 ± 20.88 ^ab^	303.88 ± 69.11 ^a^	189.33 ± 26.07 ^ab^
BUN mg/dL	31.60 ± 5.39 ^a^	26.87 ± 1.77 ^ab^	22.70 ± 1.32 ^b^	23.66 ± 1.09 ^ab^	26.33 ± 1.49 ^ab^
Creatinine mg/dL	0.38 ± 0.05 ^b^	0.57 ± 0.06 ^ab^	0.71 ± 0.05 ^a^	0.67 ± 0.05 ^a^	0.55 ± 0.03 ^ab^
Cholesterol mg/dL	88.40 ± 6.45	78.37 ± 5.56	80.00 ± 3.57	87.00 ± 9.16	83.66 ± 7.46
Triglycerides mg/dL	483.60 ± 108.90	526.00 ± 39.66	608.40 ± 47.22	619.00 ± 148.98	511.11 ± 89.22
HDL mg/dL	52.53 ± 1.78	39.72 ± 3.32	46.87 ± 1.26	50.68 ± 5.05	44.18 ± 3.36
LDL mg/dL	25.40 ± 4.43	27.87 ± 2.54	26.90 ± 2.64	30.66 ± 6.53	32.67 ± 5.09

Data are presented as mean ± SE. Different lowercase letters (a,b) indicate statistical groupings determined by one-way ANOVA followed by Tukey’s post hoc multiple-comparison test. Groups sharing at least one letter are not significantly different, whereas groups with no letter in common differ significantly (*p* < 0.05). Group sample sizes were as follows: control (*n* = 5), GDM (*n* = 8), GDM + V10 (*n* = 10), GDM + V20 (*n* = 9), and GDM + V30 (*n* = 9). GDM, gestational diabetes mellitus; V10, vildagliptin 10 mg/kg; V20, vildagliptin 20 mg/kg; V30, vildagliptin 30 mg/kg; ALT, alanine aminotransferase; AST, aspartate aminotransferase; BUN, blood urea nitrogen; HDL, high-density lipoprotein; LDL, low-density lipoprotein.

**Table 2:** Effects of vildagliptin on fetal and placental growth parameters in gestational diabetes mellitus. FW, PW, PE.

	Control	GDM	GDM + V10	GDM + V20	GDM + V30
FW	1.39 ± 0.18	1.25 ± 0.04	1.02 ± 0.15	1.24 ± 0.04	1.27 ± 0.15
PW	0.43 ± 0.03	0.49 ± 0.02	0.41 ± 0.03	0.48 ± 0.03	0.46 ± 0.02
PE	3.15 ± 0.30 ^a^	2.56 ± 0.13 ^ab^	2.18 ± 0.28 ^b^	2.64 ± 0.14 ^ab^	2.91 ± 0.26 ^ab^

Data are presented as mean ± SE. Different lowercase letters (a,b) indicate statistical groupings determined by one-way ANOVA followed by Tukey’s post hoc multiple-comparison test. Groups sharing at least one letter are not significantly different, whereas groups with no letter in common differ significantly (*p* < 0.05). Group sample sizes were as follows: control (*n* = 5), GDM (*n* = 8), GDM + V10 (*n* = 10), GDM + V20 (*n* = 9), and GDM + V30 (*n* = 9). GDM, gestational diabetes mellitus; V10, vildagliptin 10 mg/kg; V20, vildagliptin 20 mg/kg; V30, vildagliptin 30 mg/kg; FW, mean fetal weight; PW, mean placental weight; PE, placental efficiency.

## Data Availability

The data presented in this study are available on request from the corresponding author.
